# Books and Literature

**DOI:** 10.2193/2009-490

**Published:** 2010-12-13

**Authors:** CARLA G HEISTER

**Affiliations:** 1 Henry Graves Memorial Library, Yale School of Forestry and Environmental Studies195 Prospect Street, New Haven, CT 06511, USA. Telephone: 203-432-5132; FAX: 203-432-5942; e-mail: carla.heister@yale.edu

## RECENT BOOKS

Barnard, S. M., ed. 2009. **Bats in Captivity.** Logos Press, Washington, D.C. 600 pp. $99.95, ISBN: 9781934899021 (cloth); $74.95, ISBN: 9781934899038 (paper).

Billingshouse, S. I., ed. 2009. **Arctic National Wildlife Refuge: Issues and Legislation.** Nova Science Publ., Hauppauge, NY. 82 pp. $43, ISBN: 9781606928059 (paper).

Chittenden H., and I. Whyte. 2009. **Roberts Bird Guide: Kruger National Park and Adjacent Lowveld: a Guide to More than 420 Birds in the Region.** Jacana Media, Johannesburg, South Africa. 264 pp. $18.95, ISBN: 9781770096387 (paper).

Columbus, A. M., and L. Kuznetsov, eds. 2009. **Endangered Species: New Research.** Nova Science Publ., Hauppauge, NY. 360 pp. $89, ISBN: 9781606922415 (cloth); $79, ISBN: 9781608764686 (ebook).

Hager, R., and C. Jones, eds. 2009. **Reproductive Skew in Vertebrates: Proximate and Ultimate Causes.** Cambridge Univ. Press, New York, NY. 546 pp. $108, ISBN: 9780521864091 (cloth).

Hanson, E. 2009. **World of Hummingbirds.** Stackpole, Mechanicsburg, PA. 156 pp. $21.95, ISBN: 9780811736060 (paper).

Hohorst, C., Jr. 2009. **Wings of Paradise: Birds of the Louisiana Wetlands.** Louisiana Univ. Press, Baton Rouge. 144 pp. $39.95, ISBN: 9780807134504 (cloth).

Kempthorne, D., and M. D. Myers. 2009. **Radar Technology Applied to Migratory Conservation and Management.** Nova Science, Hauppauge, NY. 165 pp. $43, ISBN: 9781606929353 (paper).

Kerry, K. R., and M. J. Riddle, eds. 2009. **Health ofAntarctic Wildlife: a Challenge for Science and Policy.** Springer, New York, NY. 470 pp. $309, ISBN: 9783540939221 (cloth).

Ladds, P. W. 2009. **Pathology of Australian Native Wildlife.** CSIRO, Collingwood, Victoria, Australia. 416 pp. $172, ISBN: 9780643094444 (cloth).

Link, W. A., and R. J. Barker. 2009. **Bayesian Inference: with Ecological Applications.** Academic, New York, NY. 400 pp. $62.95, ISBN: 9780123748546 (cloth).

McCracken, D. P. 2008. **Saving the Zululand Wilderness: an Early Struggle for Nature Conservation.** Jacana Media, Johannesburg, South Africa. 176 pp. $47.95, ISBN: 9781770095960 (paper).

Mellor, D., E. Patterson-Kane, K. J. Stafford. 2009. **The Sciences of Animal Welfare.** Wiley-Blackwell, Ames, IA. 224 pp. $59.99, ISBN: 9781405134958 (paper).

Menon, V. 2009. **Mammals of India.** Princeton Univ. Press, Princeton, NJ. 208 pp. $35, ISBN: 9780691140674 (paper).

Officer, C., and J. Page. 2009. **When the Planet Rages: Natural Disasters, Global Warming and the Future of the Earth.** Oxford Univ. Press, New York, NY. 248 pp. $16.95, ISBN: 9780195377019 (paper).

Owino, A. O., and J. O. Oyugi, eds. 2009. **Directory of Conservation Funding Sources for Developing Countries: Conservation Biology, Education & Training, Fellowships & Scholarships.** Nova Science Publ., Hauppauge, NY. 189 pp. $79, ISBN: 9781607413677 (cloth).

Perillo, G. M. E., E. Wolanski, D. R. Cahoon, and M. M. Brinson. 2009. **Coastal Wetlands: an Integrated Ecosystem Approach.** Elsevier, New York, NY. 974 pp. $175, ISBN: 9780444531032 (cloth).

Romero, A. 2009. **Cave Biology: Life in Darkness.** Cambridge Univ. Press, New York, NY. 306 pp. $120, ISBN: 9780521828468 (cloth); $60, ISBN: 9780521535533 (paper).

Schmidt-French, B. A., and C. A. Butler. 2009. **Do Bats Drink Blood? Fascinating Answers to Questions about Bats.** Rutgers Univ. Press, Piscataway, NJ. 176 pp. $21.95, ISBN: 9780813545882 (paper).

Scholes, R. J., and K. Mennell, eds. 2009. **Elephant Management: a Scientific Assessment for South Africa.** Witwatersrand Univ. Press, Johannesburg, South Africa. 645 pp. $79.95, ISBN: 9781868144792 (paper).

Thomson, D. L., E. G. Cooch, and M. J. Conroy, eds. 2009. **Modeling Demographic Processes in Marked Populations.** Springer, New York, NY. 1136 pp. $169, ISBN: 9780387781501 (cloth).

UNESCO. 2009. **World Heritage Sites: a Complete Guide to 878 UNESCO World Heritage Sites.** Firefly Books, Buffalo, NY. 832 pp. $29.95, ISBN: 9781554074631 (paper).

Vega, E. T., ed. 2008. **Endangered Species Act Update and Impact.** Nova Science Publ., Hauppauge, NY. 120 pp. $69, ISBN: 9781604561340 (cloth).

Voevodin, A. F., and P. A. Marx. 2009. **Simian Virology.** Wiley-Blackwell, Ames, IA. 508 pp. $199.99, ISBN: 9780813824321 (cloth).

Wilson, J. D., A. D. Evans, and P. V. Grice. 2009. **Bird Conservation and Agriculture.** Cambridge Univ. Press, New York, NY. 404 pp. $135, ISBN: 9780521571814 (cloth); $63, ISBN: 9780521734721 (cloth).

## CURRENT LITERATURE

Aongola, L., I. Liayo, D. Ndopu, S. Bass, J. Makumba, I. Nyambe, J. Chileshe, M. Maimbolwa, A. Pope, J. Daka, K. Munyinda, M. Sichilongo, B. Dalal-Clayton, and N. Munyinda. 2009. **Creating and protecting Zambia's wealth: experience and next steps in environmental mainstreaming.** 68 pp. IIED Natur. Resour. Issues 14. Available from: Earthprint, P.O. Box 119, Stevenage, Hertfordshire, SG1 4TP, England. Cost: $15. Also online at: http://www.iied.org/pubs/pdfs/ 17502IIED.pdf.

Burley, T. E., and J. D. Peine. 2009. **NBII-SAIN Data Management Toolkit.** U.S.G.S. Open-File Rep. 2009-1170. 96 pp. Available from: USGS Information Services, Box 25286, Denver, CO 80225. Also online at: http://pubs.usgs.gov/of/2009/1170/pdf/ofr2009-1170.pdf.

Casazza, M. L., C. T. Overton, M. A. Farinha, A. Torregrosa,J. P. Fleskes, M. R. Miller, J. S. Sedinger, and E. Kolada. 2009. **Ecology of greater sage-grouse in the Bi-State Planning Area Final Report, September 2007.** U.S.G.S. Open-File Rep. 2009-1113. 50 pp. Available from: USGS Information Services, Box 25286, Denver, CO 80225. Also online at: http://pubs.usgs.gov/of/2009/1113/pdf/ofr20091113.pdf.

Gleason, R. A., B. A. Tangen, M. K. Laubhan, R. G. Finocchiaro, and J. F. Stamm. 2009. **Literature review and database of relations between salinity and aquatic biota—applications to Bowdoin National Wildlife Refuge, Montana.** U.S.G.S. Open-File Rep. 2009-5098. 76 pp. Available from: USGS Information Services, Box 25286, Denver, CO 80225. Also online at: http://pubs.usgs.gov/sir/2009/5098/pdf/sir2009-5098.pdf.

Holmes, B. E. 2008. **A review of black-footed ferret reintroduction in northwest Colorado, 2001—2006.** BLM Tech. Note. 426. 43 pp. Available online at: http://www.blm.gov/nstc/library/pdf/TN426.pdf.

Montag, J. M., and H. M. Stinchfield. 2009. **Moosehorn National Wildlife Refuge workbook summary.** U.S.G.S. Open-File Rep. 20091167. 28 pp. Available from: USGS Information Services, Box 25286, Denver, CO 80225. Also online at: http://pubs.usgs.gov/of/2009/1167/ pdf/OF09_1167.pdf.

Nussear, K. E., T. C. Esque, R. D. Inman, L. Gass, K. A. Thomas, C. S. A. Wallace, J. B. Blainey, D. M. Miller, and R. H. Webb. 2009. **Modeling habitat of the desert tortoise *(Gopherus agassizii*) in the Mojave and parts of the Sonoran Deserts of California, Nevada, Utah, and Arizona.** U.S.G.S. Open-File Rep. 2009-1102. 18 pp. Available from: USGS Information Services, Box 25286, Denver, CO 80225. Also online at: http://pubs.usgs.gov/of/2009/1102/pdf/ofr20091102.pdf.

Partridge, S., T. Smith, and T. Lewis. 2009. **Black and brown bear activity at selected coastal sites in Glacier Bay National Park and Preserve, Alaska: a preliminary assessment using noninvasive procedures.** U.S.G.S. Open-File Rep. 2009-1169. 62 pp. Available from: USGS Information Services, Box 25286, Denver, CO 80225. Also online at: http://pubs.usgs.gov/of/2009/1169/pdf/ofr20091169.pdf.

Reed, R. N., and G. H. Rodda. 2009. **Giant constrictors: Biological and management profiles and an establishment risk assessment for nine large species of pythons, anacondas, and the boa constrictor.** U.S.G.S Open-File Rep. 2009—1202. 302 pp. CD-ROM. Available from: USGS Information Services, Box 25286, Denver, CO 80225. Also online at: http://pubs.usgs.gov/of/2009/1202/pdf/OF09-1202.pdf.

Ryberg, K. R., and G. Hiemenz. 2009. **Summary and analysis of water- quality data for the Arrowwood National Wildlife Refuge, east-central North Dakota, 1987—2004.** U.S.G.S. Sci. Invest. Rep. 2009—5017. 92 pp. + suppls. Available from: USGS Information Services, Box 25286, Denver, CO 80225. Also online at: http://pubs.usgs.gov/sir/2009/5017/ pdf/sir2009-5017.pdf.

Sexton, N. R., N. Burkardt, M. E. Swann, and S. C. Stewart. 2009. **Stakeholder evaluation of Canaan Valley National Wildlife Refuge- completion report.** U.S.G.S. Open-File Rep. 2009-1030. 66 pp. Available from: USGS Information Services, Box 25286, Denver, CO 80225. Also online at: http://pubs.usgs.gov/of/2009/1030/pdf/OF09-1030.pdf.

Slonecker, E. T., L. E. Milheim, and P. R. Claggett. 2009. **A landscape indicator approach to the identification and articulation of the consequences of land cover change in the Mid-Atlantic region, 19732001.** U.S.G.S. Open-File Rep. 2009-1187. 41 pp. Available from: USGS Information Services, Box 25286, Denver, CO 80225. Also online at: http://pubs.usgs.gov/of/2009/1187/of2009-1187.pdf.

U.S. Fish and Wildlife Service. 2009. **Desert national wildlife refuge complex: Ash Meadows, Desert, Moapa Valley, and Pahranagat National Wildlife Refuges: final comprehensive conservation plan and environmental impact statement.** 2 vol. Available online at: http:// www.fws.gov/desertcomplex/ccp.htm.

Watson, R. T., M. Fuller, M. Pokras, and W. G. Hunt, eds. 2009. **Ingestion of lead from spent ammunition: implications for wildlife and humans.** 394 pp. Peregrine Fund. Available from: The Peregrine Fund, 5668 W. Flying Hawk Lane, Boise, ID 83709. Cost: $25 + $6 shipping; Also online at: http://www.peregrinefund.org/Lead_ conference/2008PbConf_Proceedings.htm.

## WEBSITES—ZOONOSES

**Zoonoses and veterinary public health—World Health Organization** http://www.who.int/zoonoses/en/

**CDC Emerging Infectious Diseases—online journal** http://www.cdc.gov/ncidod/EID/index.htm **Zoonotic Diseases Tutorial—University of Wisconsin-Madison** http://www.vetmed.wisc.edu/pbs/zoonoses/

**Zoonotic Diseases Section of Institutional Animal Care and Use Committee—University of California-Santa Barbara** http://research.ucsb.edu/iacuc/zoonotic.shtml

